# A Nearctic-Neotropical Migratory Songbird’s Nesting Phenology and Clutch Size are Predictors of Accumulated Cyclone Energy

**DOI:** 10.1038/s41598-018-28302-3

**Published:** 2018-07-02

**Authors:** Christopher M. Heckscher

**Affiliations:** Delaware State University, Department of Agriculture and Natural Resources, 1200N. DuPont Highway, Dover, Delaware, 19901 USA

## Abstract

The breeding season phenology of Nearctic-Neotropical migratory songbirds is constrained by subsequent seasons resulting in single-brooded behavior (one successful clutch per year) in some species. Early cessation of the nesting season prior to an active hurricane season will allow for behavioral plasticity during a physiologically challenging migration. Hurricane activity shows a high degree of inter-annual variability. I show that a single-brooded Nearctic-breeding species’ (*Catharus fuscescens*) nesting phenology and clutch size are significant predictors of Accumulated Cyclone Energy. The most skilled predictive model includes both mean clutch initiation date and mean clutch size (*R*^2^ = 0.84). Spearman rank correlation coefficients for both predictors with subsequent major hurricanes (1998–2016) are −0.55 and 0.52, respectively. Therefore, May and June clutch initiation and clutch size showed stronger correlations with subsequent hurricanes than early season (prior to August) meteorological predictions widely publicized by CSU, NOAA, and TSR (≤0.45, 2003–2014). Rainfall anomalies in the southern Amazon basin associated with ENSO cycles are a possible proximate cue affecting phenology and clutch size. This discovery potentially has far-reaching ornithological, meteorological, and social implications and shows that tropical storms significantly constrain breeding season behavior providing renewed evidence that hurricane activity is a primary factor regulating Nearctic-Neotropical migratory songbird populations.

## Introduction

North Atlantic hurricane activity varies greatly from year to year. Because of the potential social and economic ramifications of active hurricane seasons the search for long-range predictors of hurricane activity has been ongoing for nearly forty years^1–3^. Since 1984, various long- and short-range predictors have been used by meteorologists in an attempt to produce ever more skillful predictive models^1–4^. Long-range forecasts allow coastal communities in hurricane prone regions time to prepare for active hurricane seasons^5^. Currently, the El Niño- southern oscillation (ENSO) and the Atlantic multidecadal oscillation are the most influential climate phenomena used to predict Atlantic hurricane seasons^5^. Three predictive models are annually publicized to inform the public: Colorado State University (CSU), NOAA, and Tropical Storm Risk (TSR).

Since 1950, 84% of all hurricanes and 93% of all major hurricanes in the Atlantic basin have formed in August through October during peak southbound songbird migration^5,6^. Hurricanes pose serious threats to migratory birds that must navigate from North America to Central and South America^7,8^. Hurricanes can cause direct songbird mortality by forcing birds into the water, blowing them off course and out to sea, exposing them to extreme weather conditions such as high winds and torrential downpours, or destruction of surrounding habitat which can leave them vulnerable to predation^7,9^. Indirect effects may include forcing birds into sub-optimal habitat at a time when rapid refueling is critically important to complete migration^8^. Butler (2000) provided data showing that some eastern songbird species’ populations fluctuate in response to the severity of tropical weather suggesting that in at least some species there is high mortality associated with severe tropical storms.

The Veery (*Catharus fuscescens*) is a thrush (Aves: Turdidae) that migrates annually from northern North America to South America crossing the Gulf of Mexico and the Caribbean Sea in September and October during peak hurricane season^10–12^. The timing of arrival in South America is largely determined by the timing of breeding season activity: the date of clutch completion in May and June is a significant predictor of the entry date into South America the subsequent autumn^11^. Like several Nearctic-Neotropical migrant songbirds, Veeries are single-brooded (i.e., one successful clutch per year) due to the temporal demands of subsequent seasons^11,13,14^. However, reproductively unsuccessful females will re-nest until molt initiation which is thought to occur at the point when prolonging reproductive effort becomes too costly due to seasonal carry over effects^11^. Timely arrival of birds on non-breeding grounds is important due to competition for resources^15^. Individuals that prolong breeding-season activity as a consequence of repeated nest failure presumably have less time to migrate and arrive later in South America^11^. En route consequences may include less time to refuel at stopovers or wait out inclement weather conditions^16–18^.

Veery breeding behavior has been studied at a Delaware, USA, breeding site since 1998. Inevitably, each season a subset of females will fail to fledge young^11^. Recognizing that reproductively unsuccessful female Veeries cease re-nesting attempts earlier in some years than others, and that reproductive phenology has a direct effect on the timing of migration^11,17^, I tested the hypothesis that early cessation of the nesting season (i.e., molt initiation) occurs in years with a propensity for high hurricane activity. I reasoned that early cessation of the nesting season might allow flexibility in migratory behavior allowing birds to delay migration during storm events or circumnavigate storms without compromising arrival date in South America. Understanding also that climate can affect avian physiology and subsequent clutch size^19,20^ and that clutch size tends to decrease as the breeding season progresses^21^, I tested for a relationship between clutch size and subsequent hurricane activity. If nesting phenology is significantly constrained by active hurricane seasons and clutch sizes are affected by environmental conditions preceding those seasons then both should be predictors of hurricane activity.

## Results and Discussion

**Figure 1:**
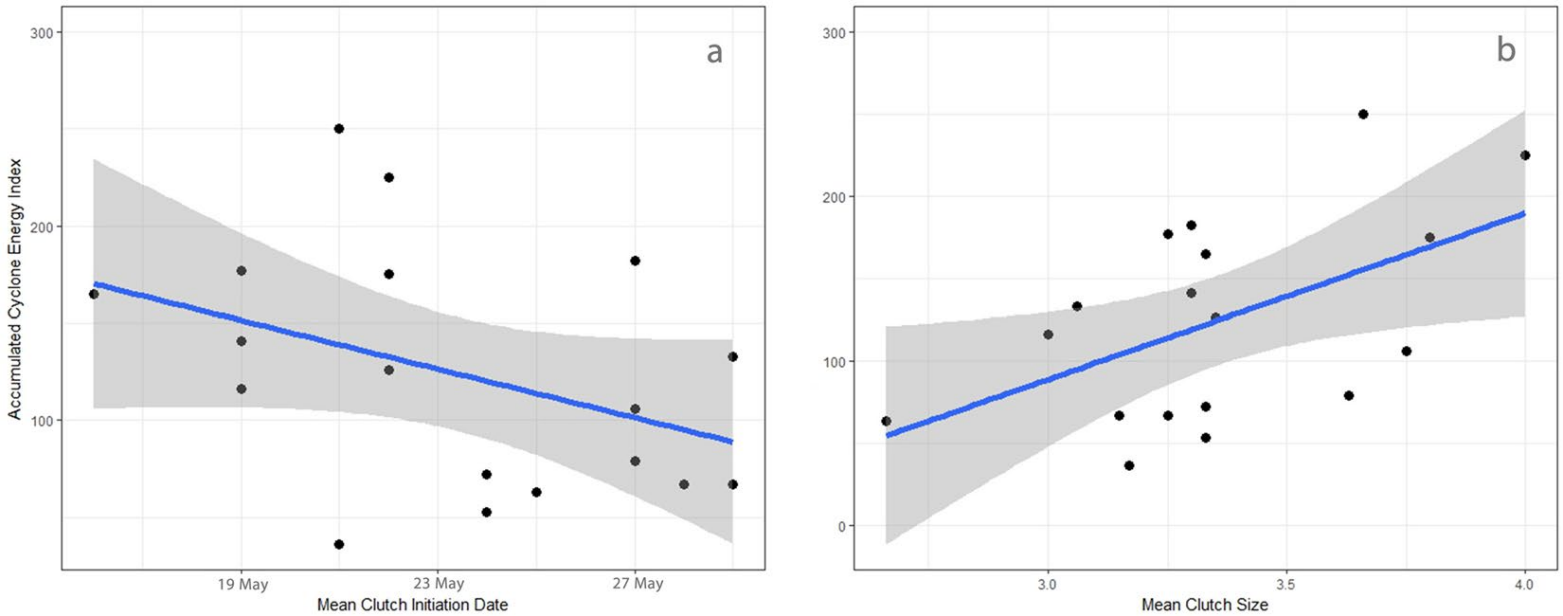
Relationship between two avian life history predictors and subsequent Atlantic basin Accumulated Cyclone Energy (ACE). (**a**) Relationship between mean clutch initiation date and ACE for the period 1998 to 2016, excluding 2008 (y = −8.2x + 306998), (**b**) Relationship between mean clutch size and ACE for the period 1998 to 2016, excluding 2008 (y = 144x − 353.2). Shaded area represents 95% confidence interval boundaries. Data are from a Delaware, USA, Veery population.

The Accumulated Cyclone Energy (ACE) index is generally thought to be the best measure of seasonal hurricane activity^5^. ACE takes into account the strength and duration of all named storms in a season. During the period 1998 to 2016 (excluding 2008) the Delaware Veery population’s mean clutch initiation date and clutch size were significant predictors of subsequent Atlantic basin ACE (Table 1, Fig. 1). Using clutch initiation and clutch size as multiple predictors, regression analysis resulted in a highly significant predictive model (Table 1). In addition, the number of major hurricanes (≥Category 3) produced in a season is of great societal and meteorological interest. Clutch initiation date and clutch size were correlated with both the number of subsequent major hurricanes and ACE. Spearman rank correlation coefficients for clutch initiation and average clutch size show *r-ranks* of −0.55 and 0.52 for major hurricanes, respectively, and 0.45 for average clutch size and ACE (−0.36 for clutch initiation and ACE). Therefore, May and June songbird phenology and clutch size were equal or more accurate long-range predictors of hurricane activity than the early season (prior to August) forecasts annually publicized by CSU, NOAA and TSR (*r-ranks* ≤ 0.45)^5^. However, it should be noted that although the years of the avian data encompass the meteorological models (2003–2014) the latter includes a smaller sample size^5^.

**Table 1:** Results of linear and multiple linear regression showing the relationship between two avian life history predictors (mean clutch initiation date and clutch size) and subsequent Atlantic basin Accumulated Cyclone Energy.

Model	*β*	CI	*R* ^*2*^	*F*	DF	P-value
Clutch initiation	−8.2	−11.7, −0.03	0.28	6.2	1, 16	0.02
Mean clutch size	144.5	28.4, 173.4	0.79	59.3	1, 16	<0.0001
Clutch initiation × Mean clutch size	289938	16452, 563424	0.84	39.2	2, 15	<0.0001
*Clutch initiation*	−7.7	−10.7, −0.02				
*Mean clutch size*	132	33.1, 180.2				

For each model, the slope parameter (*β*), confidence interval (CI), coefficient of determination (*R*^*2*^), F-statistic (*F*), degrees of freedom (DF), and probability values are given. Confidence intervals (95%) are the result of bootstrapping using 1000 replicates. P-values represent the probability of obtaining the F-ratio given the distribution of the F-statistic and the degrees of freedom if the null hypothesis of no relationship is true. Individual terms for the multiple regression model are italicized. Data are from a Delaware, USA, Veery population.

Veery clutch size declines as the breeding season progresses (1998–2016: *r*^2^ = 0.08, *P* = 0.001; y = −4.2x + 4.4; *n* = 123); however, seasonal progression explains little of the variation. In addition, mean clutch initiation date and mean clutch size were not correlated (Pearson’s *r* = −0.07, *P* = 0.77) confirming that the relationship between date and clutch size is not straightforward. Conversely, there is a strong relationship between mean clutch size and hurricane activity (Fig. 1). These data are likely due in part to a lack of small clutches that are typical of late season attempts as males abandon parental care earlier than females to initiate molt resulting in a decrease in clutch size late in the season^11^. However, the strength of the relationship between clutch size and ACE also implies that environmental conditions conducive to facilitating active hurricane seasons (perhaps including conditions on non-breeding grounds)^19^ plays a role in directly regulating clutch size such that females produce larger clutches on average prior to active hurricane seasons.

**Figure 2:**
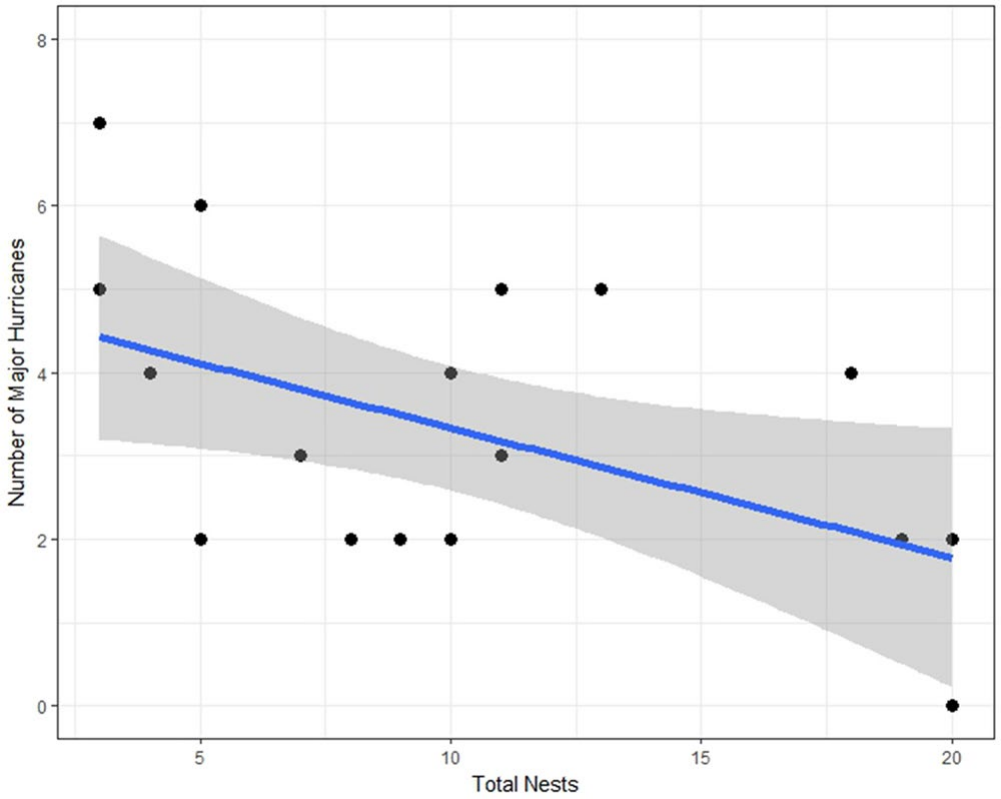
Relationship between the total number of Veery nests found in a 180 ha study site and the number of major Atlantic hurricanes (≥Category 3) the subsequent season (y = −0.05x + 1.7). Shaded area represents 95% confidence interval boundaries. Data are from a Delaware, USA, breeding site 1998 to 2016.

These results reveal that Veeries terminate the breeding season early in years more likely to produce major tropical storms. Early molt initiation allows individuals to begin migration earlier^17^ which may allow them to delay migration during storm events or circumnavigate storms without compromising arrival date in South America. In addition, early termination of the breeding season will allow birds more time to prepare for a physiologically challenging migration^19,22^. Conversely, in years less likely to produce major hurricanes females can extend reproductive activity later in the season without compromising fitness. For example, only three females have been known to have attempted a second brood in this 19-year study population and all three did so prior to seasons of below average tropical cyclone activity including the only year (2013) in which no major hurricanes occurred in the Atlantic basin: 2013 (*n* = 1), 2015 (*n* = 2). When mean clutch initiation date occurred after 23 May (*n* = 9), 77% of those seasons produced ≤2 major Atlantic hurricanes; however, when mean clutch initiation date was prior to 23 May (*n* = 9) 89% produced ≥3 major hurricanes (*χ*^2^ = 4.8, df = 1, *P = *0.03). To corroborate that nest construction was inhibited by active hurricane seasons (i.e., re-nesting attempts ceased earlier in active hurricane years resulting in fewer nests initiated in the study area), I examined the relationship between the total nests found at the study site each season and future major hurricanes (1998–2016). There was a significant relationship (*r*^2^ = 0.25, *P* = 0.04; Fig. 2) corroborating that reduced sample size in the dataset is biologically meaningful and indicative of active hurricane seasons. Thus, the total number of nests found (proportional to total nests constructed) in a fixed area of forest over time is also a significant predictor of future hurricane activity, independent of nest phenology and clutch size.

For decades, ornithologists have been searching for proximate factors that explain how Nearctic-Neotropical migratory songbird populations are regulated^23,24^. Loss and fragmentation of breeding and wintering habitat have been primary culprits and more recently habitat loss or degradation at migratory stopover sites^23,25,26^. Although a combination of contributing factors is probable, strong evidence of a primary causal factor regulating fluctuations in songbird populations has not emerged. Migratory periods are the seasons of greatest risk for Nearctic-Neotropical migrants^27,28^. Most attribute that to predation or habitat loss and degradation of stopover sites^26,29^. Butler (2000) provided support for the “storm hypothesis” which posits that populations of songbirds breeding in eastern North America are negatively associated with severe tropical storm events due to mortality during autumn migration. Of 14 species including Veery, Butler (2000) found direct correlations between abundance and prior storms in only 3 but suspected that some species might show a lag time of multiple years after population decline before recovery potentially masking relationships. Considering results herein, and those of Butler (2000), it appears that for species crossing the Gulf of Mexico and Caribbean Sea that the development of meteorological conditions conducive for active Atlantic hurricane seasons is a primary factor regulating populations, not only resulting in direct mortality during storm events, but also by significantly constraining breeding season activity. Given that reproductive phenology fluctuates annually in relation to ACE then tropical storm activity is likely to have been the most consistent source of selective pressure (i.e., mortality) during autumn migration and perhaps the entire annual cycle.

Veeries from eastern and western North America are sympatric on wintering grounds and the timing of their Gulf of Mexico and Caribbean Sea crossings overlap^10,12^ so it seems probable that the species has been affected by tropical storms similarly range-wide. Of the meteorological predictors known, there are several that might influence most of the North American Veery population. For example, the annual North Atlantic Oscillation (NAO) index has also been used to predict the probability of hurricane activity^30^. Strong negative NAO events affect temperature in eastern North America and are associated with an increased probability of major hurricanes affecting the Gulf Coast (NOAA National Center for Environmental Information: https://www.ncdc.noaa.gov/teleconnections/nao/). In addition, sea level pressure in the Gulf and Caribbean Sea during April and May, precisely the time Veeries are traversing these bodies of water, is also a significant predictor of subsequent Atlantic hurricane activity. These and other predictors are possible factors affecting Veeries. For example, songbirds are sensitive to changes in atmospheric pressure^31^. However, ENSO cycles have dramatic effects on the climate of the tropics including the Amazon basin^32^. Veeries spend much of the non-breeding season concentrated in the southern Amazon basin^10,12^. In negative ENSO (La Niña) years, there is a strong correlation between latitude and rainfall with southern areas of the basin, in general, receiving greater amounts than northern portions of Amazonia^32^. La Niña is a significant predictor of Atlantic hurricane activity: in La Niña years there is a high probability of one or more hurricanes affecting areas traversed by songbirds migrating to the tropics^4^. Such tropical rainfall anomalies are thought to positively affect food resources which are known to translate to effects on avian physiology, survival, and breeding season success providing a link between a known hurricane predictor and avian physiology^28,33^. Black-throated Blue Warblers (*Setophaga caerulescens*) spend the Nearctic winter in the Greater Antilles. Sillett *et al*. (2000) found a correlation between annual survival and recruitment of second-year Black-throated Blue Warblers breeding in New Hampshire and former La Niña events as measured by the Southern Oscillation Index (SOI). In Veery, I found a non-significant inverse relationship between the percent of the population consisting of second-year birds and mean monthly SOI the preceding Dec through May, 1998–2016 (*r*^2^ = 0.17, *P*-value = 0.08; y = −0.5x + 24.4), consistent with higher return rates (higher percentage of after-second-year birds) in La Niña years. Higher return rates in La Niña years coupled with the adjustment of breeding season phenology and clutch size (i.e., rainfall anomalies resulting in increased physiological condition and larger clutches on average) may partially offset the effect of increased mortality during major storm events the following autumn. Ultimately this may result in masking direct correlations between abundance and major storm events perhaps explaining the mixed results found by Butler (2000). Therefore, it appears these processes (i.e., a constrained breeding season, increased clutch size perhaps due to physiological condition, and a propensity for higher return rates in La Niña years) are linked via large-scale meteorological events (i.e., ENSO cycles) and act in synergy resulting in a complex relationship between avian behavior and global climate cycles which may have adaptive consequences in terms of partially alleviating the effect of increased mortality in active hurricane seasons.

Many temperate-breeding songbird species cross into the Neotropics in September and October. The effect of tropical storms on breeding behavior reported herein may not be restricted to Veery and perhaps extends to non-passerines such as raptors. Identifying species whose reproductive biology is similarly constrained and the proximate environmental cues they may use for adaptive response should be a research priority bearing in mind that large-scale climate indices (e.g., ENSO, NAO, SOI) are not always appropriate to examine effects on small scales (e.g., discrete populations) as their environmental influence can vary geographically^34^.

As global climate changes, an increase in the intensity of hurricanes coupled with a decrease in cyclone translation speed are projected which suggests compounding long-term negative effects on songbird populations^35–37^. In combination with other stressors (e.g., increased degradation or loss of habitat at breeding, wintering, or stopover sites), this may exacerbate songbird declines over the next century through both increased mortality and constraints on breeding season behavior. Songbird breeding behavior could help predict the severity of Atlantic hurricane seasons. Future research should focus on confirming constraints on additional Veery populations and whether other Nearctic-Neotropical migrant birds are similarly affected. High priority should be placed on identifying the environmental cues used to adjust breeding season activity as it is not clear whether the effects reported herein are the result of a known hurricane predictor - undiscovered meteorological teleconnections are possible, the discovery of which could improve the accuracy of long-range predictive models.

## Methods

The study took place in Delaware, USA, at White Clay Creek State Park (39.7382°N, 75.7598°W). The site is approximately 180 ha and mostly second-growth and mature temperate broadleaf forest with rolling hills and stream valleys ~50–105 m elevation. From 1998 through 2016, individual Veeries were affixed with unique color band combinations. Nest searching was initiated the first week of May and was conducted by 1–3 observers daily for ~4–7 hrs, 4–6 days wk ^−1^ (dependent on weather) until the third week of July when Veeries no longer exhibit breeding behavior^38^. Nest searching effort remained constant within each field season and identical search techniques were maintained whereby an observer remained vigilant for Veery activity (song, calls, flights, foraging) while slowly traversing the study area. Nests were found by following adults carrying food or nesting material to nests or by flushing females from nests. After a nest failed, there was a concerted effort to locate each female’s re-nesting attempt by revisiting those territories periodically until the third week of July.

Statistical analyses were accomplished using the R statistical package version 3.4.4 (R Development Core Team 2015, Vienna, Austria). Data were initially examined to confirm normality and check for collinearity (Pearson’s correlation). Multiple stages of the breeding season were correlated so I chose clutch initiation date as a predictor to represent breeding season phenology. When necessary, I estimated clutch initiation date by backtracking from a known date of a subsequent nesting event (i.e., clutch completion, hatch date, fledge date). Veery lay one egg per day and nestlings can be reliably aged shortly after hatching^21^. I used annual mean clutch size from all nests with a known completed clutch for each year. I used linear regression to model clutch initiation date and clutch size separately with the response variable ACE. Multiple linear regression was used to model the predictor variables with ACE simultaneously. Because mean values were used for regressions I used a weight term (1/SE) to account for variance in the data and variable sample size. To check fit, I bootstrapped each model using 1000 replicates with the R-package ‘boot’^39^. A post-hoc generalized linear regression using a poisson distribution and log link function was used to test the relationship between total nests found annually and the subsequent number of major hurricanes. The SOI was used to predict the percent of yearling birds (second-year) in the population each year using a post hoc generalized linear regression. Mean monthly SOI values were those from the preceding December to May and were taken from the Australian Government Bureau of Meteorology (http://www.bom.gov.au/climate/current/soihtm1.shtml). All seasonal hurricane data were obtained from the National Hurricane Center Annual Summaries (available at http://www.nhc.noaa.gov/data/tcr/). Hurricane categories were those established by the Saffir-Simpson Hurricane Wind Scale and are used by the National Hurricane Center (http://www.nhc.noaa.gov/aboutsshws.php).

Spearman rank correlation coefficients were used to compare *r-ranks* of avian predictors to those of meteorological models^5^. With the exception of total nests found, data for 2008 were excluded as clutch initiation date could not be projected for any nest found that year (i.e., because all nests discovered failed before clutch initiation or were found mid-way through the nestling phase and failed prior to fledging). For nest initiation and clutch size, this resulted in 131 and 150 nests used in analysis over an 18 year period, respectively ($\bar{x}$ = 7.3, range 3–16 yr^−1^; $\bar{x}$ = 8.3, range 3–17 yr^−1^; Supplementary Table S1). To test the difference in the number of hurricanes before and after 23 May I used a post-hoc Chi-square goodness of fit test. The Delaware State University Institutional Animal Use and Care Committee approved all research methods and all methods were in accordance with that committee. All required state and federal permits were obtained.

### Data Availability

The data analyzed during the current study appear in the Supplementary Material or are otherwise available upon reasonable request from the author.

## Electronic supplementary material

Dataset 1
