# Supplementary material for: A Nearctic-Neotropical Migratory Songbird’s Nesting Phenology and Clutch Size are Predictors of Accumulated Cyclone Energy
Source: Sci Rep. 2018 Jul 2;8:9899. doi: 10.1038/s41598-018-28302-3 (PMC6028460; doi:10.1038/s41598-018-28302-3)
Supplement: Supplementary file 1 — Dataset 1 [file 41598_2018_28302_MOESM1_ESM.doc]

SUPPLEMENTARY MATERIAL

**A Nearctic-Neotropical Migratory Songbird’s Nesting Phenology and Clutch Size are Predictors of Accumulated Cyclone Energy**

**Tracking Number: SREP-17-47846B**

**Christopher M. Heckscher**

**Delaware State University**

**Department of Agriculture and Natural Resources**

**1200 N. DuPont Highway**

**Dover, Delaware 19901**

Supplementary Table S1.

Supplementary Table S1. Data from a Delaware, USA, Veery breeding population (1998 – 2016, excluding 2008) used for significant predictors in linear and multiple regression to model subsequent North Atlantic hurricane activity.

|  |  |  | | |  |  |  |  |  | | |  |
| --- | --- | --- | --- | --- | --- | --- | --- | --- | --- | --- | --- | --- |
|  |  | Clutch Initiation Date | | |  |  |  |  | Clutch Size | | |  |
|  |  |  | | |  |  |  |  |  | | |  |
|  |  | |  |  |  |  |  |  | |  |  | |
| Year | N (proportion of nests found) | | Mean | Range | ± Standard Error |  | N | Mean | | Range | ± Standard Error | |
|  |  | |  |  |  |  |  |  | |  |  | |
| 1998 | 5 (0.71) | | 27 May | 15 May – 28 June | 16.1 |  | 6 | 3.30 | | 2 - 4 | 1.1 | |
| 1999 | 12 (0.92) | | 19 May | 13 May – 29 May | 5.2 |  | 12 | 3.25 | | 2 - 4 | 0.6 | |
| 2000 | 11 (1.0) | | 19 May | 9 May – 12 June | 10.8 |  | 11 | 3.00 | | 2 - 4 | 0.7 | |
| 2001 | 4 (1.0) | | 26 May | 17 May – 14 June | 11.3 |  | 4 | 3.75 | | 3 - 5 | 0.8 | |
| 2002 | 6 (0.66) | | 27 May | 19 May – 3 June | 5.3 |  | 8 | 3.25 | | 2 - 4 | 0.7 | |
| 2003 | 5 (0.71) | | 22 May | 17 May – 3 June | 5.9 |  | 5 | 3.80 | | 3 - 4 | 0.4 | |
| 2004 | 4 (0.8) | | 22 May | 10 May – 16 June | 14.6 |  | 4 | 4.00 | | 4 | 0 | |
| 2005 | 3 (1.0) | | 21 May | 19 May – 25 May | 2.6 |  | 3 | 3.66 | | 3 - 4 | 0.5 | |
| 2006 | 5 (0.63) | | 27 May | 18 May – 26 May | 3.2 |  | 8 | 3.63 | | 2 - 4 | 0.7 | |
| 2007 | 3 (0.6) | | 24 May | 24 May – 1 June | 5.8 |  | 3 | 3.33 | | 3 - 4 | 0.5 | |
| 2009 | 8 (1.0) | | 24 May | 15 May – 6 June | 9.1 |  | 6 | 3.33 | | 3 - 4 | 0.5 | |
| 2010 | 6 (0.6) | | 16 May | 13 May – 9 May | 2.1 |  | 5 | 3.33 | | 3 - 4 | 1.1 | |
| 2011 | 13 (0.72) | | 22 May | 14 May – 16 June | 10.1 |  | 12 | 3.35 | | 3 - 4 | 0.9 | |
| 2012 | 12 (0.63) | | 29 May | 11 May – 25 June | 16.1 |  | 15 | 3.06 | | 2 - 4 | 0.9 | |
| 2013 | 16 (0.84) | | 21 May | 12 May – 18 June | 8.3 |  | 17 | 3.17 | | 2 - 5 | 1.3 | |
| 2014 | 8 (0.4) | | 26 May | 15 May – 17 June | 9.9 |  | 12 | 3.15 | | 2 - 4 | 1.2 | |
| 2015 | 6 (0.6) | | 25 May | 15 May – 22 May | 4.8 |  | 9 | 2.66 | | 2 - 3 | 0.5 | |
| 2016 | 4 (0.4) | | 19 May | 17 May – 21 May | 1.8 |  | 10 | 3.30 | | 2 - 4 | 0.6 | |
|  |  | |  |  |  |  |  |  | |  |  | |
|  |  | |  |  |  |  |  |  | |  |  | |
